# Development of a Cost-Effective and Food-Grade Medium for Rice Cellular Agriculture

**DOI:** 10.3390/foods15071203

**Published:** 2026-04-02

**Authors:** Moeto Matsumoto, Keisuke Igarashi

**Affiliations:** Graduate School of Agricultural Science, Tohoku University, Sendai 980-8572, Japan; matsumoto.moeto.q8@dc.tohoku.ac.jp

**Keywords:** cellular agriculture, plant cellular agriculture, rice, food-grade medium, cost reduction, FG-N6CI

## Abstract

The global challenge of feeding a growing population while minimizing environmental impacts necessitates novel food production systems. Plant cellular agriculture offers a sustainable alternative for producing food ingredients; however, its commercial viability is hindered by the high costs and regulatory hurdles associated with conventional reagent-grade culture media. In this study, we developed a novel, cost-effective, and food-grade basal culture medium, FG-N6CI, for rice cellular agriculture. By replacing reagent-grade basal-medium components of the N6CI medium with food-grade alternatives, specifically by substituting chemical reagents with yeast extract, kelp powder, manganese yeast, and a boron supplement, we formulated a food-grade basal nutrient composition while retaining reagent-grade phytohormones. Rice (*Oryza sativa* L. ‘Taichung 65’) callus cultured on FG-N6CI medium exhibited significantly higher fresh weight (7.1 g) than the conventional N6CI medium (5.8 g) after 35 days (*p* < 0.05). Gene expression analysis showed no significant differences between the expression of *OsHDA710* and *OsTIR1*, suggesting that FG-N6CI supports normal cellular proliferation and signaling similar to the standard medium. Economically, the cost of FG-N6CI medium was reduced by approximately 72% compared with that of the commercial reagent-grade mixture (219 JPY/L vs. 795 JPY/L). These results demonstrate that FG-N6CI is an economically competitive basal medium for scaling-up plant cellular agriculture.

## 1. Introduction

The global population continues to expand at an unprecedented rate, and contemporary food production systems are increasingly unable to meet this rising demand. According to the United Nations, the global population is projected to reach approximately 8.5 billion by 2030 and 9.7 billion by 2050, before peaking at around 10.4 billion in the 2080s [1]. This sustained demographic growth inevitably increases the demand for food, disrupting the equilibrium between existing production capacity and global requirements. Furthermore, environmental factors, including global warming and natural disasters, have diminished agricultural yields, exacerbating the instability of the global food supply [2].

Over the past several decades, agricultural production has intensified to meet the rapidly growing food demand, resulting in a substantial increase in the environmental burden. Current food systems face critical sustainability challenges, with greenhouse gas emissions and water pollution substantially affecting the global environment [3,4]. There is a detrimental interplay between population growth and environmental issues. Specifically, efforts to intensify agriculture in order to boost yields often result in environmental pollution and ecosystem degradation, creating a vicious cycle that undermines the long-term sustainability of food production. To address these challenges, there is an urgent need to develop innovative food production technologies that go beyond traditional agricultural frameworks.

Recently, “cellular agriculture” has attracted considerable attention as a novel production paradigm capable of tackling these issues. This field involves the indoor production of cellular biomass or specific extracts for food and industrial applications by culturing cells derived from living organisms [5].

In plant cell research, it has been demonstrated that culturing cells of *Coffea arabica* and roasting the harvested biomass can produce a beverage base. Although it exhibits fewer nutty and earthy notes characteristic of the conventional coffee beans, it possesses distinct aromatic profiles, such as caramelized sugar and caramel, resulting from the high sugar content of the raw material [6,7]. Furthermore, cell cultures of *Rubus* species (e.g., cloudberries and lingonberries) have been reported to contain higher levels of protein, dietary fiber, and essential amino acids than their whole-fruit counterparts, offering both high nutritional value and favorable palatability. Although nutrient-dense foods such as macroalgae are typically classified as “superfoods,” these findings suggest that cellular agriculture could serve as a novel and sustainable source of such superfoods [8].

Ensuring product safety is a critical challenge in the social implementation of these technologies as viable food production systems. Currently, definitive international standards and regulatory frameworks for the safety assessment of cellular agricultural products have yet to be finalized; instead, regulatory authorities in various countries are in the process of developing their own independent systems. For example, the U.S. Food and Drug Administration (FDA) proposed a joint regulatory framework with the USDA Food Safety and Inspection Service (USDA-FSIS). The FDA evaluates the entire process, from cell collection to proliferation and differentiation, requiring the submission of rigorous data concerning cell line stability, all culture medium components, impurities generated during manufacturing, and comprehensive hazard analyses [9]. Similarly, the Centre for Food Safety (CFS) in Hong Kong has established more specific management criteria for media components. The CFS requires explicit identification and purity standards for antimicrobials, growth factors, and modifying agents added to the media. Furthermore, it mandates toxicological evidence to demonstrate that the residual levels of any non-food-grade components pose no harm to human health [10].

These regulatory trends highlight the challenges of directly repurposing reagent-grade medium components, traditionally used in tissue engineering and biopharmaceutical development, for food production. While previous studies on plant cell culture medium optimization have largely focused on nutrient composition or cost efficiency using reagent-grade chemicals, the present study takes a distinct approach by systematically replacing each component of the N6CI medium according to its regulatory classification under Japanese food additive standards. This regulatory-grade substitution framework-substituting only components not approved as food additives while retaining authorized components-constitutes a “safe-by-design” strategy that reduces both material costs and future regulatory burdens for commercial-scale plant cellular agriculture. This study aimed to reconcile cost reduction with safety assurance in plant cellular agriculture, thereby establishing a feasible pathway for commercial scale-up and social implementation.

## 2. Materials and Methods

In the present study, a food-grade (FG) basal medium was formulated by substituting the essential components of conventional plant cell culture media with food-grade raw materials. Subsequently, plant cell cultures were performed using the developed medium, and their performance was evaluated by comparing callus fresh weight and gene expression profiles with those of conventional media.

The N6 medium, developed by Chu et al. in 1975, is a widely recognized standard basal medium for plant cell culture [11]. It is noteworthy that the “N6CI medium” used as the positive control in this study is not the unmodified N6 basal medium; rather, it is an in-house modification of the N6 medium, supplemented with casamino acids and proline to optimize rice callus induction. Hereafter, this variant is referred to as “N6CI medium.” The addition of casamino acids and proline is known to significantly enhance the proliferation and regeneration efficiency of cultured rice cells [12] and has been established as the standard optimized protocol in our laboratory. Therefore, to rigorously validate the practical utility of the FG medium as a viable alternative, its performance was compared with that of the optimized N6CI medium, rather than the standard N6 formulation.

Basal media provide a comprehensive set of nutrients essential for plant cell proliferation, categorized as macronutrients, micronutrients, vitamins and organic supplements, and carbon source (Table 1). For practical culture applications, these basal formulations were supplemented with appropriate types and concentrations of phytohormones tailored to the specific plant species.

### 2.1. Design of FG-N6CI (Food-Grade Basal Medium)

In this experiment, the FG-N6CI basal medium was designed based on the composition of the conventional N6CI basal medium. The components of the FG-N6CI basal medium were selected by substituting traditional reagents with either food-grade additives or food ingredients or by omitting them entirely, depending on their regulatory status.

The suitability of each N6CI component as a food additive was verified according to “Japan’s Specifications and Standards for Food Additives, 10th Ed. (2024)” [13] and is listed by the Japan Food Chemical Research Foundation [14,15]. Based on this regulatory review, the components were categorized into three groups: “Authorized,” “Conditionally Authorized,” and “Unauthorized.”

The FG-N6CI basal medium was formulated using food additives for “Authorized” and “Conditionally Authorized” components, whereas “Unauthorized” components were either substituted with general food ingredients or excluded. However, the phytohormones added to the basal medium were of reagent grade.

Four components—MnSO_4_, H_3_BO_3_, KI, and Casamino acids—were identified as unauthorized food additives. Suitable food-grade alternatives were selected based on their physiological roles in the culture medium as follows:

MnSO_4_ serves as the primary source of manganese in plant tissues. It was replaced with manganese-enriched edible yeast (Medience Corporation, Tokyo, Japan) as a food-grade manganese source. The composition of manganese yeast is provided in Table A1.

H_3_BO_3_ functions as a boron source. For its substitution, a boron supplement, “Boron 3 mg Veg Capsules” (NOW Foods, Bloomingdale, IL, USA), containing calcium borogluconate (CaBG) as the primary ingredient, was utilized. Each capsule (370 mg) contained 3 mg boron (NOW Foods). Nutritional profiles of the supplements are presented in Table A2.

KI provides both potassium and iodine. Commercially available kombu (kelp) powder was selected as a natural source of these elements. Typical kombu contains approximately 225 mg of iodine per 100 g [16], and the required amount of kombu powder was calculated based on reported concentrations [16].

Casamino acids, a mixture of amino acids and small peptides obtained from the acid hydrolysis of casein, provide essential nitrogen and amino acids in plant cell cultures. A previous study reported that substituting casamino acids with yeast extract during the vegetative growth of kuritake mushrooms resulted in improved yields and growth rates [17]. Based on this precedent, the yeast extract “Hi-Max GL” (Fuji Foods Corporation, Yokohama, Japan) was selected as a food-grade alternative to supply both amino acids and nitrogen simultaneously. Its composition is provided in Table A3.

### 2.2. Medium Composition

FG-N6CI (experimental group)

The experimental group comprised food additive-grade macro-elements (KNO_3_, (NH_4_)_2_SO_4_, MgSO_4_·7H_2_O, KH_2_PO_4_, CaCl_2_·2H_2_O), trace elements (FeSO_4_·7H2O, ZnSO_4_·7H_2_O, EDTA·2Na), and vitamins/amino acids (*myo*-inositol, niacin, vitamin B6, vitamin B1, glycine, and proline). Food ingredients were used as substitutes for the unauthorized components, as described above. Sucrose (sugar) and agar “Kanten Cook” (Ina Food Industry, Ina, Japan) were of food grade. The pH was adjusted to 5.8 with food-grade KOH (Table 2) (detailed sourcing is presented in Table A4).

N6CI (positive control)

The positive control comprised reagent-grade chemicals. Basal salts were supplied as Chu’s N6 Basal Salt Mixture (PhytoTech Labs, Lenexa, KS, USA), which contains the same macro- and micronutrient ions as listed in Table 1 at equivalent concentrations. The vitamins, amino acids (casamino acids, proline, and glycine), sucrose, and agar were of reagent grade. The pH was adjusted to 5.8 with reagent-grade KOH (Table 3) (see Table A5 for detailed sourcing).

NC (negative control)

The negative control consisted of water, plant hormones (2,4-D), and food-grade agar without added nutrients. The pH of the NC medium was intentionally left unadjusted, as the absence of buffering components would render pH adjustment unstable, and the addition of pH-adjusting agents (KOH or HCl) would introduce exogenous ions that compromise the nutrient-free nature of the negative control. The 2,4-D concentration used (2 mg/L, ~9 μM) is insufficient to meaningfully alter the pH of the aqueous solution.

All media contained 2.0 mg/L 2,4-D and were autoclaved at 121 °C, 220 kPa for 20 min.

### 2.3. Plant Materials and Culture

Rice seeds (*Oryza sativa* L. ‘Taichung 65’) were dehusked, surface-disinfected with 5% (*v*/*v*) sodium hypochlorite solution containing approximately 0.1% (*v*/*v*) Tween 20 as a surfactant for 30 min, and then washed with sterile water. Seeds were placed on the N6CI medium to induce callus formation (28 °C, 15,000 lux continuous white fluorescent light [PHCbi MLR-352-PJ, Tokyo, Japan; Light step 4LS]). To ensure initial callus homogeneity, induced calli were first maintained in reagent-grade N6CI medium for approximately four weeks. The resulting calli were then divided and subcultured onto the three test media (FG-N6CI, N6CI, and NC) at a standardized initial fresh weight of 0.1 g per dish (*n* = 13).

### 2.4. Measurements

Callus fresh weight (FW) was measured every 7 days for 35 days. Statistical analyses were performed using Python (version 3.11) with the scipy (version 1.16.3) and statsmodels (version 0.14.6) libraries. For fresh weight data, one-way ANOVA followed by Tukey’s HSD test was applied (*p* < 0.05). One replicate of the FG-N6CI group (FG12) was excluded from analysis at days 21, 28, and 35 due to missing measurements (*n* = 12 for FG-N6CI at those timepoints; *n* = 13 for all other groups and timepoints). For gene expression data, group differences were assessed using a two-tailed unpaired Student’s *t*-test. For gene expression analysis, total RNA was extracted using the RNeasy Plant Mini Kit (Qiagen, Hilden, Germany), treated with DNase, and reverse-transcribed using SuperScript II (Thermo Fisher, Waltham, MA, USA). RT-qPCR was performed using TB Green Premix Ex Taq II (Takara Bio, Kusatsu, Japan) with three technical replicates per biological replicate. Each reaction (20 μL total) contained 10 μL TB Green Premix Ex Taq II (2×), 0.4 μM each primer, and 1 μL cDNA template, using a standard two-step cycling protocol (95 °C 30 s; 40 cycles of 95 °C 5 s, 60 °C 30 s), followed by melt-curve analysis to confirm single-product amplification. The expression of *OsHDA710* (histone deacetylase 710; Os02g0215200), which reflects epigenetic stability of cultured cells, and *OsTIR1* (transport inhibitor response 1; Os05g0150500), a key auxin receptor central to callus induction and proliferation, was normalized to *Actin7* expression using the efficiency-corrected relative quantification method (Pfaffl method [18]). Amplification efficiencies were determined from standard curves (*Actin7*: E = 2.267; *OsHDA710*: E = 2.117; *OsTIR1*: E = 2.003). Primers were designed using Primer3 (v.0.4.0) with amplicon size 100–200 bp, Tm 58–62 °C, and GC content 40–60%. The primers used are listed in Table A6.

### 2.5. Cost Calculation

The costs (JPY/L) were calculated based on manufacturer catalog prices or actual purchase prices as of 2023–2025. For components received as manufacturer samples, list prices were obtained directly from the manufacturers. Supplier names and product details are provided in Table A4 and Table A5. For N6CI, calculations were made for both “mixture-based” (using commercial salt mix) and “reagent-based” (individual chemicals) preparations.

## 3. Results

### 3.1. Callus Proliferation

**Figure 1:**
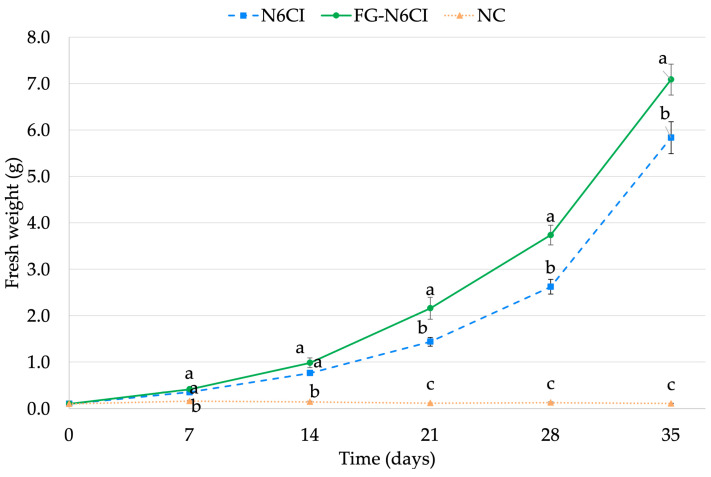
Time course changes in the fresh weight of rice calli. Data are presented as mean ± SE. N6CI and NC: *n* = 13 at all timepoints. FG-N6CI: *n* = 13 at days 0, 7, and 14; *n* = 12 at days 21, 28, and 35 due to one missing replicate (FG12). Different letters indicate significant differences among groups at each timepoint (one-way ANOVA followed by Tukey’s HSD test, *p* < 0.05). No statistical test was applied at day 0, as all replicates had an identical starting weight of 0.1 g.

Figure 1 illustrates the time course of the changes in callus fresh weight under each condition. In the NC medium, almost no increase in callus weight was observed throughout the 35-day cultivation period. In contrast, both the FG-N6CI and N6CI media exhibited remarkable increase in fresh weight as the cultivation period progressed. The growth curves for these two media showed a clear divergence around day 21, with FG-N6CI demonstrating a superior growth rate than N6CI. At the final point (day 35), the average fresh weight of FG-N6CI reached 7.1 g, which was significantly higher than the 5.8 g observed in the N6CI medium. This result suggests that FG-N6CI promotes callus proliferation more effectively than the N6CI medium.

### 3.2. Callus Morphology

**Figure 2:**
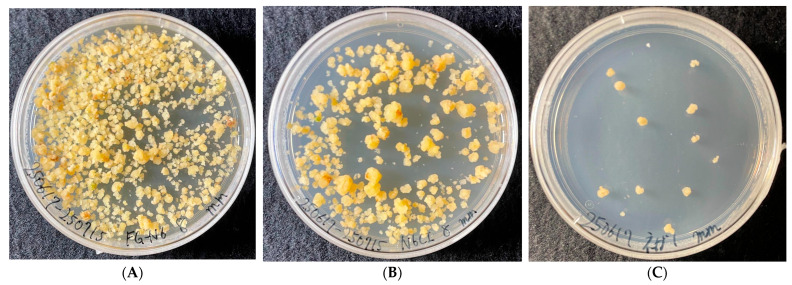
Morphological observation of rice calli after 35 days of cultivation. (**A**) Rice calli cultured in FG-N6CI (food-grade). (**B**) Rice calli cultured in the N6CI medium (reagent-grade positive control). (**C**) Rice calli cultured in the NC medium (negative control without basal nutrients).

Morphological observations of calli after 35 days of culture are shown in Figure 2. Distinct differences were evident among the treatment groups. In FG-N6CI, the calli appeared pale yellow and friable and densely covered the surface of the plastic culture dish, demonstrating the most vigorous growth of the three conditions (Figure 2A). In the N6CI medium, yellow granular callus masses were observed growing at a moderate density (Figure 2B). In the NC medium, only a few microscopic callus fragments were observed across the plastic culture dish, indicating that proliferation was almost completely suppressed (Figure 2C). These morphological findings are consistent with the fresh weight data described above.

### 3.3. Gene Expression Analysis

Sufficient amounts of RNA were obtained from calli cultured in N6CI and FG-N6CI; however, calli from the NC medium yielded only trace amounts of RNA, which were insufficient for cDNA synthesis. Therefore, RT-qPCR analysis was performed to compare the gene expression levels between the N6CI and FG-N6CI groups. The analysis targeted two genes involved in callus formation: *OsHDA710* (histone deacetylase 710 [Os02g0215200]) and *OsTIR1* (transport inhibitor response 1 [Os05g0150500]). Gene expression levels are presented as relative values, with those of calli cultured in N6CI medium set at 1. Data are shown as mean ± standard error (SE).

**Figure 3:**
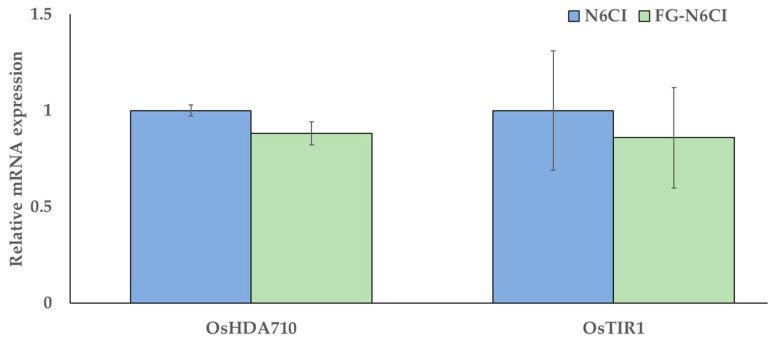
RT-qPCR analysis of *OsHDA710* and *OsTIR1* expression in rice calli after 21 days of cultivation. Relative expression levels were calculated using the efficiency-corrected relative quantification method (Pfaffl method), normalized to the internal control gene *Actin7*, and presented relative to those of the N6CI medium group (set to 1.0). Data represent the mean ± standard error (SE) of three biological replicates, each measured in triplicate (three technical replicates per biological replicate). No significant differences were observed between FG-N6CI and N6CI (*OsHDA710*: *p* = 0.147; *OsTIR1*: *p* = 0.745; Student’s *t*-test, two-tailed).

The expression of *OsHDA710* in calli cultured in FG-N6CI showed a lower mean value than that in the N6CI medium, but no significant difference was observed. Similarly, although the expression of *OsTIR1* in the FG-N6CI group was slightly lower than that in the N6CI group, the difference was not statistically significant (Figure 3). These results suggest that calli cultured in FG-N6CI maintained expression levels of the selected marker genes-reflecting epigenetic stability (*OsHDA710*) and auxin-signaling competence (*OsTIR1*)-comparable to those cultured in the standard reagent-grade N6CI medium, indicating no major perturbation of these specific molecular pathways under food-grade culture conditions.

### 3.4. Comparison of the Costs of Culture Media

Costs were calculated based on manufacturer catalog prices or actual purchase prices as of 2023–2025. For components received as manufacturer samples, list prices were obtained directly from the manufacturers. For the N6CI basal medium used as a reference, costs were estimated for two scenarios: preparation using individual reagent components and preparation using the commercially available premixed salt powder utilized in this study. The raw material costs per liter (JPY/L) are listed in Table 4. All values were rounded to two decimal places, and components with calculated costs of less than 0.01 JPY are indicated as “<0.01”.

The cost of the N6CI basal medium prepared using the commercial premixed powder was approximately 795 JPY/L, whereas that of FG-N6CI was approximately 219 JPY/L, representing a 72% cost reduction. Even when compared with the version prepared with individual reagents (approximately 571 JPY/L), FG-N6CI achieved a 62% reduction in cost.

## 4. Discussion

### 4.1. High Nitrogen-Utilizing Capacity of Rice

Rice is adapted to anaerobic paddy fields and excels in utilizing ammonium nitrogen (NH_4_^+^) [19]. Although high ammonium levels can be toxic to many plants, causing rhizosphere acidification and cation competition [20], rice efficiently takes up ammonium via OsAMT transporters and assimilates it into amino acids [21,22]. N6CI uses reagent-grade ammonium sulfate. Successful growth on FG-N6CI confirms that food-additive-grade ammonium sulfate provided an equally effective ammonium supply without toxicity.

### 4.2. Impact of Specific Food Ingredients (Molecular Perspectives)

Because multiple components were substituted simultaneously in FG-N6CI, it is not possible to attribute the observed growth enhancement to any single substitution. The following discussion presents mechanistic hypotheses for each substitution individually; their combined effects may be additive, synergistic, or partially redundant, and future studies using single-substitution designs will be necessary to isolate the contribution of each component. The superior growth observed in FG-N6CI likely resulted from the active molecular components present in the food-grade substitutions.

#### 4.2.1. Yeast Extract and Energy Savings via Salvage Pathway

Casamino acids (in N6CI) provide a balanced nitrogen source. The substitute yeast extract (Hi-Max GL) contains glutamate, nucleotides (IMP/GMP), and NaCl [23]. We hypothesize that glutamate may act as a signaling molecule in root apical meristems and promote cell division [24] and that the nucleotides provided by yeast extract may confer metabolic advantages. The de novo synthesis of purine nucleotides in plants requires a complex 12-step reaction that consumes substantial amounts of ATP. In contrast, nucleotides provided by yeast extract may potentially be directly recycled via the salvage pathway mediated by enzymes such as hypoxanthine-guanine phosphoribosyl transferase (HGPRT) [25]. This bypass may allow cells to save substantial energy during the rapid division phase, potentially channeling resources into biomass accumulation. We acknowledge, however, that Hi-Max GL provides a more limited amino acid profile compared to casamino acids. We propose that the GS/GOGAT cycle for efficient glutamate assimilation, combined with de novo amino acid biosynthesis supported by adequate nitrogen from ammonium sulfate and glutamate, may compensate for the reduced amino acid diversity. The precise mechanism of amino acid compensation has not been experimentally validated in this study and represents a topic for future investigation.

#### 4.2.2. Kelp (Kombu) and Phytohormone Synergy

Kelp replaced KI with iodine/potassium, but likely provided secondary benefits. Seaweed extracts are biostimulants that may contain trace levels of natural cytokinins (e.g., zeatin riboside) and auxin-like compounds. The IAA content of commercial seaweed extracts has been a subject of considerable uncertainty; older bioassay-based studies reported much higher values, while modern UPLC-MS/MS quantification of commercial *Ascophyllum nodosum* extract reveals that IAA and other phytohormones are present at trace levels [26,27]. Seaweed (kelp) extracts are known to contain trace levels of IAA and other phytohormone-like compounds [27], which may contribute to callus growth through indirect modulation of endogenous phytohormone pathways rather than through direct delivery of exogenous hormones. While both media contained 2,4-D (synthetic auxin), N6CI essentially operated as an “auxin-only” system. The addition of kelp to FG-N6CI may have introduced trace cytokinins, potentially shifting the hormonal balance from a pure auxin state to a synergistic auxin-cytokinin state. This balance is known to be more effective for inducing rapid cell division [28,29].

#### 4.2.3. Manganese Yeast and Transporter Availability

MnSO4 releases free Mn^2+^, which can be problematic in complex media. Free Mn^2+^ competes with Fe^2+^ and Zn^2+^ for uptake via natural resistance-associated macrophage proteins (NRAMP) or zinc-regulated/iron-regulated transporter-like protein (ZIP) transporters [30,31,32]. Additionally, free Mn^2+^ is susceptible to oxidation to unavailable forms, such as MnO_2_ [32]. In contrast, in manganese-enriched yeast, manganese may be chelated intracellularly by proteins or polyphosphates, which potentially protects it from oxidation [33]. Plants possess yellow stripe-like (YSL) transporters specifically designed to take up metal–organic complexes [30]. We hypothesize that the chelated manganese in FG-N6CI may bypass competitive ion channels (NRAMP/ZIP) and be more efficiently absorbed via the YSL pathway, ensuring stable micronutrient availability. Furthermore, manganese-enriched yeast (Manganese yeast 5%, Medience Corporation) contains significant amounts of protein (45.2%), carbohydrates (33.6%), and lipid (2.9%) in addition to manganese (5.4%) (Table A1). These macromolecular components may provide additional nitrogen, carbon, and energy sources that potentially contribute to callus growth beyond the role of manganese alone. Disentangling these contributions from the other yeast-derived and kelp-derived components is not possible within the current experimental design and represents a limitation of this study.

#### 4.2.4. Boron Supplement and Molecular Mimicry

Calcium borogluconate (CaBG) was used as the source of boron. In biological systems, boron is typically transported not as free boric acid, but as a complex with sugar alcohols (e.g., boron sorbitol) [34]. CaBG is a complex of boron and gluconic acid (a sugar acid). This structure may exhibit “molecular mimicry” of the natural boron-sugar transport complexes, potentially allowing it to be recognized and transported more efficiently by plant boron transporters and facilitating phloem mobility [34] compared to inorganic boric acid. Such efficient delivery may support cell wall synthesis and division.

### 4.3. Mechanism Underlying Callus Morphology (Friable vs. Compact)

FG-N6CI induced friable calli (ideal for liquid suspension), whereas N6CI induced compact calli. Several factors may contribute to this difference.

Gelling Agent Hardness: Food-grade agar (FG-N6CI) may have produced a physically harder gel than reagent-grade agar (N6CI). Hard media can restrict passive water uptake and increase resistance to cell expansion, which may force proliferating cells to separate rather than adhere, resulting in a friable texture [35,36]. However, as noted in Section 4.2.2, kelp-derived phytohormone-like compounds may exert an equal or greater influence on callus texture through modulation of the auxin-to-cytokinin balance; the relative contribution of physical (gelling agent) versus biochemical (hormone balance) factors cannot be resolved from the current data.

Implications for Scaling: Friable calli are technically superior for industrial-scale applications. They disperse easily in liquid suspension bioreactors, ensuring uniform nutrient access and preventing the formation of large necrotic centers, which are common in compact clumps [37].

### 4.4. Economic and Regulatory Advantages

This 72% cost reduction demonstrates that food-grade substitution is economically effective. Beyond material costs, FG-N6CI reduces significant “regulatory costs”. Using unapproved reagents (e.g., unverified MnSO_4_ or KI) for food production would require extensive safety testing, such as GLP toxicity studies, potentially costing millions of dollars and years [38]. FG-N6CI, composed entirely of food ingredients (as the basal medium), inherently bypasses these hurdles. In jurisdictions such as the US or Singapore, this “Safe by Design” approach significantly diminishes the barrier to regulatory approval [38], accelerating the path to social implementation.

Although 2,4-D is not a food-grade ingredient, its use in plant cell culture does not inherently disqualify the resulting biomass from food safety considerations if residual levels in the final product are appropriately controlled. The present study focused on establishing a food-grade basal nutrient medium; the replacement of synthetic phytohormones with natural alternatives (e.g., natural auxins or cytokinin-active compounds derived from food sources) represents an important future direction toward a fully food-grade culture system.

## 5. Conclusions

We developed “FG-N6CI,” a food-grade basal medium for rice cellular agriculture, which achieved 72% cost reduction and superior growth (7.1 g vs. 5.8 g) compared to standard N6CI. The successful application of FG-N6CI is not solely attributable to elemental substitution, but likely arises from the specific molecular benefits of food-grade ingredients, including energy savings via the nucleotide salvage pathway (yeast extract), hormonal synergy (kelp), chelated nutrient uptake (manganese yeast), and molecular mimicry of natural transport forms (boron supplement). This study demonstrates that food-grade basal media can match or outperform reagent-grade standards, offering a scientifically robust and economically viable medium for sustainable food production.

## 6. Patents

A patent application concerning the preparation and use of FG-N6CI described in this work has been filed. A Japanese patent application (Application No. JP2025-137089) has been filed by Tohoku University, with Author M.M. and Author K.I. listed as inventors.

## Figures and Tables

**Table 1:** Composition and Regulatory Status of the N6CI Basal Medium Components.

Components	Concentration (mg/L)	Regulatory Status
**Macronutrients**		
Potassium nitrate (KNO_3_)	2830	△
Ammonium sulfate ((NH_4_)_2_SO_4_)	463	○
Potassium dihydrogen phosphate (KH_2_PO_4_)	400	○
Magnesium sulfate heptahydrate (MgSO_4_·7H_2_O)	185	○
Calcium chloride dihydrate (CaCl_2_·2H_2_O)	166	△
**Micronutrients**		
Ethylenediaminetetraacetic acid disodium salt (EDTA·2Na)	37.25	○
Ferrous sulfate heptahydrate (FeSO_4_·7H_2_O)	27.85	○
Manganese sulfate tetrahydrate (MnSO_4_·4H_2_O)	4.4	✕
Boric acid (H_3_BO_3_)	1.6	✕
Zinc sulfate heptahydrate (ZnSO_4_·7H_2_O)	1.5	△
Potassium iodide (KI)	0.8	✕
**Vitamins and Organic Supplements**		
Nicotinic acid	0.5	△
Pyridoxine hydrochloride (Pyridoxine-HCl)	0.5	○
Glycine	2	○
Thiamine hydrochloride (Thiamine-HCl)	1	○
*myo*-Inositol	100	○
Casamino acids	300	✕
**Carbon Source**		
L-Proline	2827	○
Sucrose	30,000	○

Note: ○ = Authorized (approved for use as a food additive). △ = Conditionally authorized. ✕ = Not authorized.

**Table 2:** Composition of FG-N6CI.

Components	Grade	Concentration (mg/L)
KNO_3_	Food additive	2830
(NH_4_)_2_SO_4_	Food additive	463
KH_2_PO_4_	Food additive	400
MgSO_4_·7H2O	Food additive	185
CaCl_2_·2H_2_O	Food additive	166
EDTA·2Na	Food additive	37.25
FeSO_4_·7H_2_O	Food additive	27.85
Manganese-enriched yeast	Food additive	20.04
Boron supplement (CaBG)	Nutrient supplement	34.534
ZnSO_4_·7H_2_O	Food additive	1.5
Kelp	Food	265.83
Niacin (Nicotinic acid)	Nutrient supplement	1.5
Vitamin B6 supplement (Pyridoxine-HCl)	Nutrient supplement	2.15
Glycine	Food additive	2
Vitamin B1 supplement (Thiamine-HCl)	Nutrient supplement	4.167
*myo*-inositol	Nutrient supplement	100
Yeast extract	Food additive	300
L-Proline	Food additive	2827
Table sugar	Food	30,000
2,4-D	Reagent	2
Agar	Food	8000
KOH	Food additive	q.s. to pH 5.8

In FG-N6CI, the boron supplement (CaBG) replaces boric acid entirely and is not used in addition to it.

**Table 3:** Composition of N6CI medium.

Components	Grade	Concentration (mg/L)
Chu’s N6 Basal Salt mixture	Reagent	4000
Nicotinic acid	Reagent	0.5
Pyridoxine-HCl	Reagent	0.5
Glycine	Reagent	2
Thiamine-HCl	Reagent	1
*myo*-inositol	Reagent	100
Casamino acids	Reagent	300
L-Proline	Reagent	2827
Sucrose	Reagent	30,000
2,4-D	Reagent	2
Agar	Reagent	8000
KOH	Reagent	q.s. to pH 5.8

**Table 4:** Cost composition of the N6CI basal medium and the FG-N6CI basal medium.

Components (JPY/L)	N6CI (Individual Reagents)	N6CI (Salt Mixture)	FG-N6CI
	Reagent	Reagent	Food
KNO_3_	18.40	-	16.41
(NH_4_)_2_SO_4_	1.63	-	4.17
MgSO_4_·7H_2_O	0.49	-	0.67
KH_2_PO_4_	1.60	-	2.08
CaCl_2_·2H_2_O	0.76	-	0.73
H_3_BO_3_	<0.01	-	-
Boron supplement	-	-	0.86
KI	0.06	-	-
Kelp	-	-	2.76
MnSO_4_·4H_2_O	0.03	-	-
Manganese-enriched yeast	-	-	0.24
ZnSO_4_·7H_2_O	0.08	-	0.01
FeSO_4_·7H_2_O	0.10	-	0.12
Na2·EDTA	0.63	-	0.22
N6 basal salt mixture	-	230.00	-
*myo*-inositol	5.30	5.30	2.11
Nicotinic acid	0.03	0.03	-
Niacin	-	-	0.11
Pyridoxine HCl	0.05	0.05	-
Vitamin B6 supplement	-	-	0.28
Thiamine HCl	1.69	1.69	-
Vitamin B1 supplement	-	-	0.37
Glycine	0.01	0.01	<0.01
Casamino acids	6.06	6.06	-
Yeast extract	-	-	1.50
L-Proline	285.53	285.53	76.33
Sucrose	92.40	92.40	-
Table sugar	-	-	12.00
Agar	174.24	174.24	97.87
**Total Cost (JPY/L)**	**570.68**	**795.31**	**218.81**

## Data Availability

Data is contained within the article.

## Appendix A

**Table A1:** Nutritional and elemental composition of the manganese-enriched yeast.

Alternative Source	Component	Content (%)
Manganese yeast (5% Mn)	Protein	45.2
	Lipid	2.9
	Carbohydrates	33.6
	Moisture	3.3
	Ash	15.0
	Manganese	5.4
	Sodium	0.095
	Salt (as NaCl)	0.2

Data obtained from the product specification sheet provided by the manufacturer (Medience Corporation, Tokyo, Japan).

**Table A2:** Composition and ingredients of the boron supplement.

Alternative Source	Component	Content (mg/Capsule)
Boron supplement	Boron (as calcium borogluconate)	3
	Rice flour	N.D. ^1^
	Stearic acid (vegetable source)	N.D. ^1^

^1^ N.D.: Not disclosed (used as excipients). Data obtained from the product specification sheet provided by the manufacturer (NOW Foods, Bloomingdale, IL, USA).

**Table A3:** Chemical composition of the yeast extract (Hi-Max GL).

Alternative Source	Component	Content (%)
Yeast extract (Hi-Max GL)	Monosodium glutamate	21.2
	Nucleic acids (IMP-2Na + GMP-2Na) ^1^	2.0
	Sodium chloride (NaCl)	1.9
	Moisture	4.5

^1^ IMP−2Na: Disodium inosinate; GMP−2Na: Disodium guanylate. Data obtained from the product specification sheet provided by the manufacturer (Fuji Foods Corporation, Yokohama, Japan).

**Table A4:** Suppliers and Product Details of FG-N6CI Components.

Component	Supplier	Product Number/Product Name
KNO_3_	Hayashi Pure Chemical Ind., Ltd., Osaka, Japan	47007015
(NH_4_)_2_SO_4_	Junsei Chemical Co., Ltd., Tokyo, Japan	83111-2201
KH_2_PO_4_	Hayashi Pure Chemical Ind., Ltd.	47005285
MgSO_4_·7H_2_O	Hayashi Pure Chemical Ind., Ltd.	470040050
CaCl_2_·2H_2_O	Junsei Chemical Co., Ltd.	18229-2201
EDTA·2Na	Chelest Corporation, Osaka, Japan	Chelest F-NA
FeSO_4_·7H_2_O	Hayashi Pure Chemical Ind., Ltd.	47002765
Manganese-enriched yeast	Medience Corporation, Tokyo, Japan	Manganese yeast 5%
Boron supplement (CaBG)	NOW Foods	1410CA/Boron 3 mg
ZnSO_4_·7H_2_O	Kanto Chemical Co., Inc., Tokyo, Japan	58008-17
Kelp	Yamamoto Food Co., Ltd., Hiroshima, Japan	497966060007
Niacin (Nicotinic acid)	Amosea Co., Ltd., Tokyo, Japan	Niacin Premium
Vitamin B_6_ supplement (Pyridoxine-HCl)	Nutricost, Vineyard, UT, USA	Vitamin B_6_ (Pyridoxine HCl)
Glycine	Nippon Garlic Co., Ltd., Takasaki, Japan	2023120102
Vitamin B_1_ supplement (Thiamine-HCl)	Kyowa Yakuhin Co., Ltd., Osaka, Japan	Simply B_1_
*myo*-inositol	NOW Foods	0527
Yeast extract	Fuji Foods Corporation	Hi-Max GL
L-Proline	Marugo Corporation, Osaka, Japan	L-Proline
Table sugar	Wellneo Sugar Co., Ltd., Tokyo, Japan	(Cup brand) White Sugar
2,4-D	Merck, Darmstadt, Germany	D7299
Agar	Ina Food Industry Co., Ltd.	Kanten Cook
KOH	Junsei Chemical Co., Ltd.	1310-58-3

**Table A5:** Suppliers and Product Details of Conventional N6CI Medium Components.

Component	Supplier	Product Number/Product Name
Chu (N6) Basal Salt Mixture (Powder)	PhytoTech Labs	C416
Nicotinic acid	Nacalai Tesque, Inc., Kyoto, Japan	243-26
Pyridoxine HCl	FUJIFILM Wako Pure Chemical Corp., Osaka, Japan	163-05402
Glycine	FUJIFILM Wako Pure Chemical Corp.	077-00735
Thiamine HCl	FUJIFILM Wako Pure Chemical Corp.	203-00851
*myo*-inositol	FUJIFILM Wako Pure Chemical Corp.	094-00281
Casamino acid	Nihon Pharmaceutical Co., Ltd., Tokyo, Japan	393-02145
L-Proline	FUJIFILM Wako Pure Chemical Corp.	165-04605
Sucrose	FUJIFILM Wako Pure Chemical Corp.	192-00017
2,4-D	Merck	D7299
Agar	FUJIFILM Wako Pure Chemical Corp.	016-11875
KOH	Nacalai Tesque, Inc.	28616-45

**Table A6:** Primers used for RT-qPCR.

Name	Sequence (5′ to 3′)
Rac-7_F (Actin7)	ACACCGTGCCAATCTATGAAGG
Rac-7_R (Actin7)	ACAATTTCCCGTTCAGCAGTGG
OsHDA710-F	ATCTCTCAGGCAAAGGTCATGC
OsHDA710-R	ACCACCACCAAGAAGAAGCAAC
OsTIR1-F	TTGGATGTCGTCGTGCTTGTTG
OsTIR1-R	AACAGGTGTTTCATCCGGAAGC
